# The Standard Medical Manual

**Published:** 1902-03

**Authors:** 


THE STANDARD MEDICAL MANUAL. A Handbook of Practical Medicine. By ALFRED S. BURDICK, M. D., Junior Professor of Practice of Medicine, Illinois Medical College, etc., etc. Pages, 921. Illustrated. Chicago: G. P. Engelhard & Co. [Price, cloth, $4.00.

In the very beginning the author makes it clear that he lays not the slightest claim to greatness for this book; he has no hope of it becoming a standard work, and explains that its title has reference to the publication of which he is editor; and yet in spite of his modest assurance in this regard the book will compare favorably with a great many of the more pretentious works on the same subject, which have been pushed into circulation under the cloak of originality. Dr. Burdick is a very busy man with his editorial work and goes through great masses of medical literature, books and journals. From these he has collected the best material obtainable, facts and methods which his own experience as an instructor and his own judgment as a physician tell him are of value to the every-day practitioner, and the result has been the compilation and publication of this book, which in a way fills a hitherto unoccupied field, for the material it contains is in getatable shape.

Dr. Burdick gives in brief the most important facts concerning etiology, pathology, symptomatology of the diseases treated and devotes especial attention to treatment with reference to authorities. There is no fine writing between the covers; theories are not exploited; might-have-beens and may-bes and possibilities have no place in the work and the effort he has made to be thoroughly practical and up-to-date has been eminently successful. Dr. Burdick intends the manual to be of use and assistance to the man who is practising medicine in a broad sense, and for this reason the diseases of specialists have either been competently briefed or entirely omitted; and additional space given to those diseases which the doctor is most likely to run across in his every-day life, such as tuberculosis, typhoid, diphtheria, scarlatina, pneumonia, and the like.

Quite an unusual feature in a book on practice is the attention devoted to the minor ailments which are seldom taught in colleges and rarely mentioned in text-books, but which many times are the worriment of the young man in medicine. There is given a large number of prescriptions and the importance of diet, hygiene, hydrotherapy and other physiologic methods of treatment is dwelt upon at length. The articles are arranged in alphabetical order without reference to the scientific classification of disease and in an appendix are given directions for the examination of urine and stomach contents, and for the recognition of the common forms of bacteria. It is a cleverly arranged work and it deserves the success which its value as a medical book entitles it to. N.W.W.

